# Detection of E2A-PBX1 fusion transcripts in human non-small-cell lung cancer

**DOI:** 10.1186/1756-9966-32-29

**Published:** 2013-05-20

**Authors:** Min-Li Mo, Zhao Chen, Hai-Meng Zhou, Hui Li, Tomomi Hirata, David M Jablons, Biao He

**Affiliations:** 1 School of Life Sciences, Tsinghua University, Beijing 10084, China; 2 Thoracic Oncology Program, Department of Surgery, Helen Diller Family Comprehensive Cancer Center, University of California, San Francisco, CA 94115, USA; 3 Zhejiang Provincial Key Laboratory of Applied Enzymology, Yangtze Delta Region Institute of Tsinghua University, Jiaxing 314006, Zhejiang, China; 4 Department of Surgery, Division of Thoracic Surgery, Nippon Medical School, Tokyo 113-8602, Japan

**Keywords:** NSCLC, Fusion gene, E2A-PBX1

## Abstract

**Background:**

E2A-PBX1 fusion gene caused by t(1;19)(q23;p13), has been well characterized in acute lymphoid leukemia (ALL). There is no report on E2A-PBX1 fusion transcripts in non-small-cell lung cancer (NSCLC).

**Methods:**

We used polymerase chain reaction (PCR) to detect E2A-PBX1 fusion transcripts in human NSCLC tissue specimens and cell lines. We analyzed correlation of E2A-PBX1 fusion transcripts with clinical outcomes in 76 patients with adenocarcinoma in situ (AIS) and other subgroups. We compared mutation status of k-ras, p53 and EGFR in 22 patients with E2A-PBX1 fusion transcripts.

**Results:**

We detected E2A-PBX1 transcripts in 23 of 184 (12.5%) NSCLC tissue specimens and 3 of 13 (23.1%) NSCLC cell lines. Presence of E2A-PBX1 fusion transcripts correlated with smoking status in female patients (P = 0.048), AIS histology (P = 0.006) and tumor size (P = 0.026). The overall survival was associated with gender among AIS patients (P = 0.0378) and AIS patients without E2A-PBX1 fusion transcripts (P = 0.0345), but not among AIS patients with E2A-PBX1 fusion transcripts (P = 0.6401). The overall survival was also associated with status of E2A-PBX1 fusion transcripts among AIS stage IA patients (P = 0.0363) and AIS stage IA female patients (P = 0.0174). In addition, among the 22 patients with E2A-PBX1 fusion transcripts, 12 (54.5%) patients including all four non-smokers, showed no common mutations in k-ras, p53 and EGFR.

**Conclusions:**

E2A-PBX1 fusion gene caused by t(1;19)(q23;p13) may be a common genetic change in AIS and a survival determinant for female AIS patients at early stage.

## Background

Lung cancer has been the leading cause of cancer-related deaths in developed countries [1]. Non-small-cell lung cancer (NSCLC) accounts for around 80% of all lung cancer cases. Somatic events, such as point mutation, genomic rearrangements (e.g. translocation) and changes in copy number, usually cooperatively cause alterations in oncogenes, tumor-suppressor genes, and microRNA genes, and lead to the multi-step carcinogenesis [2,3]. During tumor initiation and/or progression, encoded oncogenic proteins activated by translocations or mutations can alter cell proliferation and/or apoptosis [3], resulting in transformation events. Fusion transcripts can be caused by chromosomal translocations and may occur more frequently in solid tumors than previously understood [2-4].

E2A-PBX1 fusion protein contains the transactivation domain of E2A and the DNA-binding domain of PBX1 and is generated by t(1;19)(q23;p13) translocation [5]. t(1;19) occurs in 5% of pre-B-cell acute lymphoid leukemias (ALL) in children and adults [6] and E2A-PBX1 has been widely characterized in ALL [5-15]. E2A-PBX1 can cause transformation in several cell types in vitro and induce lymphoblastic lymphomas in transgenic mice [7-9]. Target genes of E2A-PBX1 includes fibroblast growth factor (FGF)-15 [13], WNT-16 [14], and some novel genes [10], etc. Bmi-1 regulation of INK4A-ARF was required for transformation of hematopoietic progenitors by E2A-PBX1 [15]. However, there has been no report on detection of E2A-PBX1 fusion transcripts in solid tumors. In this study, we investigated into the detection of E2A-PBX1 fusion transcripts in NSCLC and compared this genetic change with three other common mutations in NSCLC (i.e. k-ras, p53 and EGFR) [16-18]. These data suggest that E2A-PBX1 fusion transcripts caused by t(1;19)(q23;p13) may be a common somatic genetic change of importance in solid tumors and E2A-PBX1 may be a novel target for prognosis and therapy in adenocarcinoma in situ (AIS) [19].

## Methods

### Patients and tissue specimens

A total of 184 patients were chosen in this study. All eligible patients without preoperative chemotherapy or radiation treatment underwent surgical resection of a primary NSCLC and had adequate mediastinal lymph node staging at UCSF between July 1997 and January 2007. Their clinical information of patients was summarized in Table 1. Information on clinical variables and patient follow-up were obtained from a prospectively maintained database including all subjects with banked tissue in the study. Patients consented to tissue specimen collection prospectively, and the study was approved by UCSF Human Research Protection Program Committee on Human Research. Tissue specimens were snap-frozen in liquid nitrogen at the time of the operation and stored in -150°C freezer.

**Table 1:** Characteristics of NSCLC patients in the study cohort

		**Total (%)**	**E2A-PBX1 positive (%)**	**E2A-PBX1 negative (%)**	**P value**	**Median overall survival (95% CI)**	**P value**
	Total	184 (100)	23 (12.5)	161 (87.5)		105.60 (55.41 ~ 155.79)	
Age							
	Mean (years)	66.9 ± 12.0	66.0 ± 11.7	67.0 ± 12.1	0.698*		
	Range (years)	25-91	39-84	25-91			
	<71	109 (100)	13 (11.9)	96 (88.1)	0.777	69.00 (43.73 ~ 94.27)	0.7069
	≥71	75 (100)	10 (13.3)	65 (86.7)		105.60 (18.53 ~ 192.67)	
Gender					0.215		
	Male	78 (100)	7 (9.0)	71 (91.0)		64.70 (NA)	0.0889
	Female	106 (100)	16 (15.1)	90 (84.9)		105.60 (57.58 ~ 153.62)	
Race					0.606		0.1430
	Caucasian	136 (100)	17 (12.5)	119 (87.5)		81.70 (52.59 ~ 110.81)	
	Asian	27 (100)	3 (11.1)	24 (88.9)		64.70 (45.79 ~ 83.61)	
	Hispanic	7 (100)	2 (28.6)	5 (71.4)		NR	
	African-American	7 (100)	0	7 (100)		NR	
	Others	7 (100)	1 (14.3)	6 (85.7)			
Smoking					0.174		0.0868
	Smoker	127 (100)	19 (15.0)	108 (85.0)		69.00 (42.36 ~ 95.64)	
	Non-smoker	53 (100)	4 (7.5)	49 (92.5)		105.60 (35.86 ~ 175.34)	
	Unknown	4 (100)	0	4 (100)			
Pack/Year (smoker)							
	Mean	41.6 ± 23.5	46.3 ± 26.7	30.9 ± 35.9	0.623*		
	Range	1-160	5-90	1-160			
Gender × Smoking					0.097		0.0258
	Male, Smoker	59 (100)	5 (8.5)	54 (91.5)	0.733^1^	56.20 (27.25 ~ 85.15)	0.0749^1^
	Male, Non-smoker	18 (100)	2 (11.1)	16 (88.9)		NR	
	Female, Smoker	68 (100)	14 (20.6)	54 (79.4)	0.048^2^	81.70 (41.68 ~ 121.72)	0.6714^2^
	Female, Non-smoker	35 (100)	2 (5.7)	33 (94.3)		105.60 (35.04 ~ 176.16)	
	Unkown	4 (100)	0	4 (100)			
Histology					0.276		0.6013
	AIS	76 (100)	17 (22.4)	59 (77.6)	0.006^3^	105.60 (57.93 ~ 153.27)	0.1208^3^
	Invasive adenocarcinoma	76 (100)	5 (6.6)	71 (93.4)		53.10 (NA)	
	Squamous cell carcinoma	18 (100)	0	18 (100)		NR	
	Carcinoid	6 (100)	0	6 (100)		NR	
	Large	4 (100)	1 (25.0)	3 (75.0)		NR	
	Others	4 (100)	0	4 (100)			
Tumor Size					0.026*		
	Mean	3.3 ± 1.9	4.1 ± 2.8	3.2 ± 1.7			
	Range	0.5-13.0	0.9-12.0	0.5-13.0			
Pathological TNM Classification							
pt	pt1	74 (100)	9 (12.2)	65 (87.8)	0.408	105.60 (NA)	0.0915
	pt2	81 (100)	9 (11.1)	72 (88.9)		69.00 (44.22 ~ 93.78)	
	pt3	8 (100)	0	8 (100)		40.20 (26.06 ~ 54.34)	
	pt4	18 (100)	4 (22.2)	14 (77.8)		30.50 (NA)	
	Unknown	3 (100)	1 (33.3)	2 (66.6)			
pn	pn0	144 (100)	18 (12.5)	126 (87.5)	0.924	105.60 (65.68 ~ 145.52)	0.0038
	pn1	19 (100)	3 (15.8)	16 (84.2)		47.80 (32.55 ~ 63.05)	
	pn2	17 (100)	2 (11.8)	15 (88.2)		45.50 (NA)	
	pn3	2 (100)	0	2 (100)		5.20 (NA)	
	Unknown	2 (100)	0	2 (100)			
pm	pm0	171 (100)	20 (11.7)	151(88.3)	0.179	105.60 (55.99 ~ 155.21)	0.2605
	pm1	12 (100)	3 (25.0)	9 (75.0)		56.20 (35.26 ~ 77.14)	
Pathological Stage					0.426		0.0167
	Stage I	119 (100)	13 (10.9)	106 (89.1)		105.60 (65.47 ~ 145.73)	
	Stage II	22 (100)	2 (9.1)	20 (90.9)		NR	
	Stage III	29 (100)	5 (17.2)	24 (82.8)		33.60 (0.00 ~ 73.11)	
	Stage IV	12 (100)	3 (25.0)	9 (75.0)		56.20 (35.26 ~ 77.14)	
	Unknown	2 (100)	0	2 (100)			
Recurrence					0.435		<0.001
	Yes	63 (100)	6 (9.5)	57 (90.5)		39.30 (30.45 ~ 48.15)	
	No	103 (100)	14 (13.6)	89 (86.4)		NR	
	Unknown	18 (100)	1 (5.6)	17 (94.4)			

* student *t* test.

1 between male smoker and male non-smoker.

2 between female smoker and female non-smoker.

3 between AIS and invasive adenocarcinoma.

AIS: adenocarcinoma in situ; NR: not reached; NA: not available.

### Cell lines

NSCLC cell lines A549, A427, H441, H838, H1975, H1650, H322, H358, H1666, H1703, H2170, H460 and H1299, acute lymphoblastic leukemia (ALL) cell line CCRF-CEM were obtained from ATCC and cultured as recommended. ALL cell line RCH-ACV was a kind gift from Dr. Mignon Loh (Department of Paediatrics, UCSF).

### RNA extraction and polymerase chain reaction (PCR)

Total RNA from cell lines and tissues were extracted using TRIzol reagent (Invitrogen) according to manufacture’s handbook. Adult normal lung total RNA was purchased at Biochain (CA). 1μg RNA was used for cDNA synthesis (BioRad). 1μL cDNA, 0.2mM for each dNTP, 0.4μM forward (5′-caccagcctcatgcacaa-3′, according to NM_003200 1398-1416) and reverse (5′-tttctccagctccgtatggt-3′, according to NM_002585 605-624) primers, magnesium with final concentration of 2mM, the PCR buffer, Q-solution and 2U Taq enzyme provided (Qiagen) were used in the first round PCR. The reaction cycles were 95°C for 5min, followed by 30 cycles of 95°C 30s, 55°C 30s, 72°C 30s, with final extension of 7min. 1μL PCR product was used in the second round PCR. The conditions were the same except forward primer (5′-gcacaaccacgcggccc-3′, according to NM_003200 1407-1423) and reverse primer (5′-ccacgccttccgctaacagc-3′, according to NM_002585 456-475). PCR products were run on 1.5% agarose gels and dyed with ethidium bromide. GAPDH was used as internal control. Sequencing was performed using PCR primers by Quintara (CA).

### DNA extraction and mutation analysis in K-ras, p53 and EGFR

Genomic DNA was extracted from snap-frozen tissue specimens using Qiagen genomic DNA purification kit. Mutations in K-ras codon 12, p53 exons 4-8, EGFR exons 19-21 were analyzed by direct sequencing as previously reported [20-22].

### Statistical analysis

The associations between the status of E2A-PBX1 fusion transcripts and clinical values were analyzed with Pearson Chi-square test and student *t* test for category variables and continuous variables, respectively. Median survival, 95% confidence intervals (CI) was calculated by Kaplan-Meier model and the log-rank test. A Cox regression model was used in AIS patients to assess the effects of E2A-PBX1 fusion transcripts, adjusted for gender, tumor stage, smoking status, race and Eastern Cooperative Oncology Group (ECOG) performance status. All p values reported were from two-sided tests. All analysis was performed by using SPSS 13.0. A p-value ≤ 0.05 was considered as significant.

## Results

### Detection of E2A-PBX1 fusion transcripts in NSCLC

**Figure 1:**
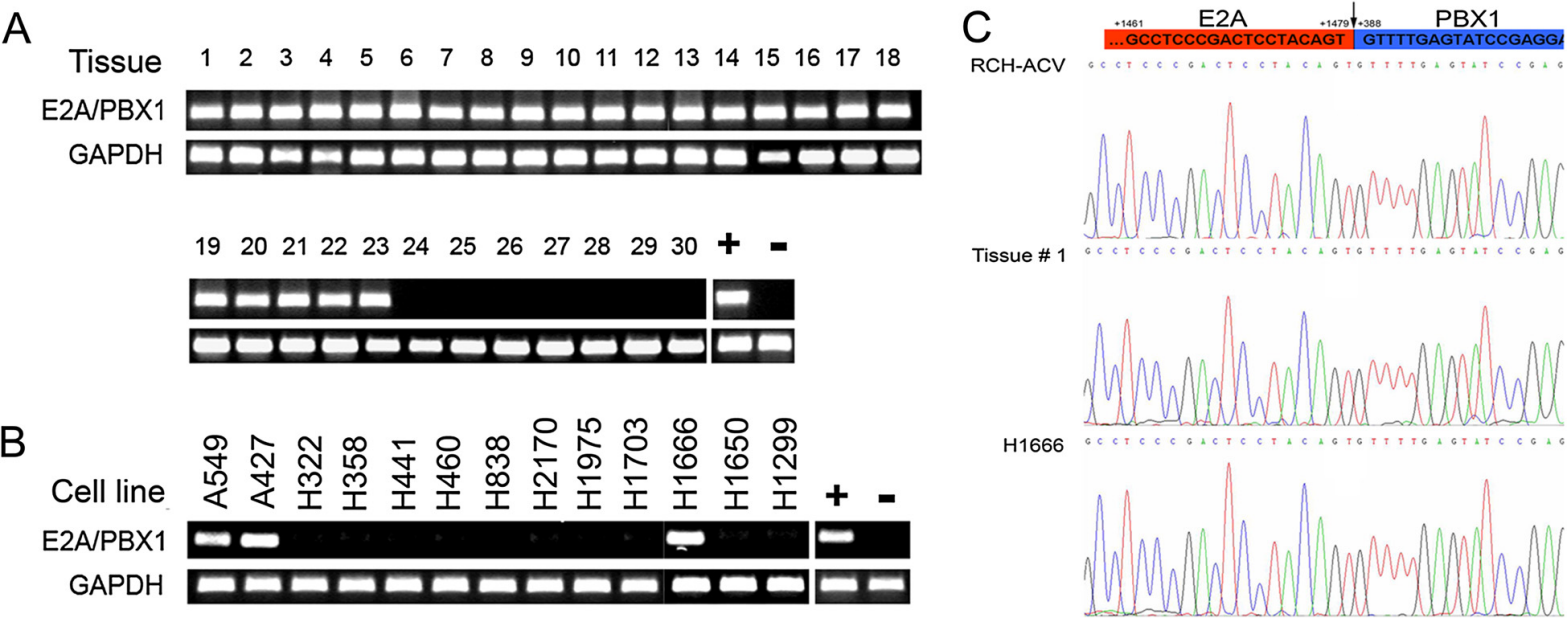
**Detection of E2A-PBX1 fusion transcripts in NSCLC.** Semi-quantitative RT-PCR in NSCLC tissues (**A**) and cell lines (**B**). GAPDH was used as internal control. RCH-ACV and CCRF-CEM were regarded as positive (marked by +) and negative (marked by -) controls, respectively. 23 positive specimens (#1-23), 6 selected negative samples (#24-29) and adult normal lung tissue (#30) were shown in (**A**). (**C**) Sequencing results of RCH-ACV, H1666 and tissue #1. Partial region around the junction site (indicated by an arrow and a dashed line) was shown. The numbers showed the positions of the sequence according to E2A (NM_003200) and PBX1 (NM_002585) mRNA sequences.

We performed nested PCR and detected E2A-PBX1 in 23/184 (12.5%) NSCLC patients as well as in positive control (RCH-ACV cell line [23,24]), but not in negative control (CEM cell line [23,24]) or adult normal lung (Figure 1A). For the 23 patients with E2A-PBX1 fusion transcripts in their tumor tissues, we did not detect the E2A-PBX1 fusion transcripts in their paired adjacent normal tissues (figures not shown). We searched the sequencing results for all the PCR products in NCBI nucleotide/translated nucleotide/protein databases by BLAST (Basic Local Alignment Search Tool). The alignments showed that all the products we obtained were human E2A-PBX1 fusion gene with 100% identities. We also detected and confirmed E2A-PBX1 fusion transcripts in 3/13 (23.1%) NSCLC cell lines (Figure 1B). Furthermore, we found that all the junction sites in these specimens were the same as that reported by Nourse J, et al. [5] (sequencing examples of the sequence around the junction site in one positive NSCLC tissue sample and cell line were was shown in Figure 1C).

### Association of E2A-PBX1 fusion transcripts with clinicopathological characteristics of NSCLC patients

We next analyzed association of the expression of E2A-PBX1 fusion transcripts and patients’ characteristics (Table 1). Smoking status was not significantly associated with the frequency of E2A-PBX1 fusion transcripts in all patients (19/127 (15.0%) in smokers and 4/56 (7.5%) in non-smokers (p = 0.174)), or in male patients (5/59 (8.5%) in smokers and 2/18 (11.1%) in non-smokers (p = 0.733). On the other hand, the frequency of E2A-PBX1 fusion transcripts in female smokers (14/68 (20.6%)) was significantly higher than that in female non-smokers (2/35 (5.7%)) (p = 0.048). The odds ratio for female smoker/non-smoker was 4.278, and 95% CI was from 0.914 to 20.026, also suggesting that the expression of E2A-PBX1 fusion transcripts correlated with smoking status among female patients with NSCLC.

The frequencies of E2A-PBX1 fusion transcripts in adenocarcinomas, squamous cell carcinomas, carcinoids and large cell carcinomas were 22/152 (14.5%), 0/18 (0%), 0/6 (0%), 1/4 (25%), respectively (p = 0.276) (Table 1). Interestingly, the frequency of E2A-PBX1 fusion transcripts in patients with AIS (17/76 (22.4%)) was significantly higher (p = 0.006) than that in patients with invasive adenocarcinoma (5/76 (6.6%)) (Table 1). The odds ratio for AIS/invasive adenocarcinoma was 4.092, and 95% CI was from 1.424 to 11.753, suggesting significant correlation between the expression of E2A-PBX1 fusion transcripts and patients with AIS. Moreover, the mean tumor size in patients with E2A-PBX1 fusion transcripts (4.1 ± 2.8cm) was significantly larger than that in patients without E2A-PBX1 fusion transcripts (3.2 ± 1.7cm) (p = 0.026) (Table 1). There was no significant association between expression of E2A-PBX1 fusion transcripts and age, gender, race, stage, or recurrence status (Table 1).

### Association of E2A-PBX1 fusion transcripts with overall survival in AIS patients

**Figure 2:**
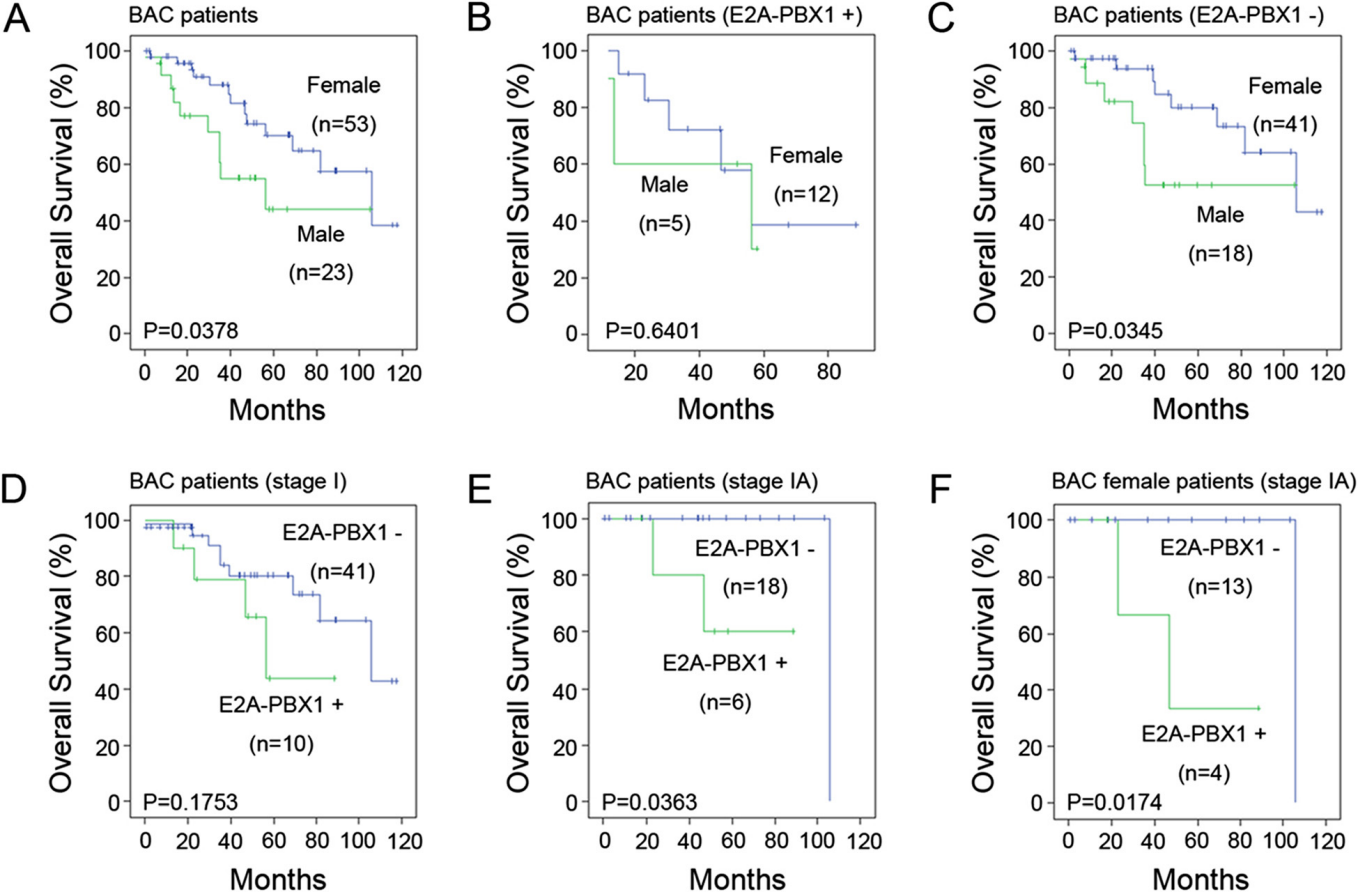
**Kaplan-Meier estimates of overall survival in AIS patients.** (**A**) 76 AIS patients, (**B**) 17 AIS patients with E2A-PBX1 fusion transcripts, (**C**) 59 AIS patients without E2A-PBX1 fusion transcripts, (**D**) 51 AIS patients at stage I, (**E**) 24 AIS patients at stage IA, and (**F**) 17 AIS female patients at stage IA. The patients were grouped by either gender (in panels **A**, **B** and **C**) or the status of E2A-PBX1 fusion transcripts (in panels **D**, **E**, and **F**).

In our study cohort of patients with AIS, females had significantly better overall survival (OS) than males (p = 0.0378; hazard ratio 0.3647; 95% CI, 0.1395 ~ 0.9532) (Table 2, Figure 2A), consistent with known data [25]. When these AIS patients were grouped by gender and expression of E2A-PBX1 fusion transcripts, no significant difference in OS was found between females and males in AIS patients with E2A-PBX1 fusion transcripts (p = 0.6401) (Figure 2B). In patients without E2A-PBX1 fusion transcripts, however, females had significantly better OS than males (p = 0.0345; hazard ratio 0.2687; 95% CI, 0.07945 ~ 0.9089) (Figure 2C). In addition, Kaplan-Meier analysis demonstrated an association between expression of E2A-PBX1 fusion transcripts and OS by stage. A statistically significant difference in OS was not observed in stage I patients (Figure 2D). OS was significantly better in E2A-PBX1 fusion transcripts (-) group than that in E2A-PBX1 fusion transcripts (+) group in stage IA patients with AIS (p = 0.0363; hazard ration 0.04104; 95% CI, 0.002065 ~ 0.8157) (Figure 2E) and female stage IA patients with AIS (p = 0.0174; hazard ration 0.02174; 95% CI, 0.0009266 ~ 0.5100) (Figure 2F). A multivariate analysis also showed that the status of E2A-PBX1 fusion transcripts (P = 0.050; hazard ratio 3.447; 95% CI, 1.002 ~ 11.857), gender (p = 0.005; hazard ratio 0.212; 95% CI, 0.071 ~ 0.628) and stage IA (p = 0.004; hazard ratio 0.011; 95% CI, 0.001 ~ 0.237) were significantly associated with overall survival.

**Table 2:** Overall survival analysis in AIS patients and subgroups

**Group**	**Gender**	**E2A-PBX1 status**	**Patient number**	**Median survival (months)**	**95% CI**	**P value**
AIS patients	Female		53	105.60	63.95 ~ 147.25	**0.0378**
	Male		23	56.20	22.34 ~ 90.06	
AIS patients with E2A-PBX1	Female		12	56.20	37.46 ~ 74.94	0.6401
	Male		5	56.20	0.00 ~ 122.80	
AIS patients without E2A-PBX1	Female		41	105.60	63.45 ~ 147.75	**0.0345**
	Male		18	NR	NA	
AIS patients		+	17	56.20	44.37 ~ 68.03	0.1235
		-	59	105.60	63.95 ~ 147.25	
AIS stage I patients		+	10	56.20	38.38 ~ 74.02	0.1753
		-	41	105.60	63.65 ~ 147.55	
AIS female patients		+	12	56.20	37.46 ~ 74.94	0.0747
		-	41	105.60	63.45 ~ 147.75	
AIS stage IA patients		+	6	NR	NA	**0.0363**
		-	18	NR	NA	
AIS stage IA female patients		+	4	46.70	8.77 ~ 84.63	**0.0174**
		-	13	105.60	NA	

NR: not reached; NA: not available.

### E2A-PBX1 fusion transcripts as a potential genetic biomarker in AIS

Mutations in K-ras, p53 and EGFR are commonly found in NSCLC [16-18]. We next screened for mutations in codon 12 of K-ras, exons 4-8 of p53, and exons 19-21 of EGFR in the specimens form patients with expression of E2A-PBX1 fusion transcripts. We also compared the status of E2A-PBX1 fusion transcripts and mutation status of K-ras, p53 and EGFR in the NSCLC cell lines used in our study (mutation information was obtained from The COSMIC (Catalogue of Somatic Mutations in Cancer) database and website [25]) (Table 3 and Table 4). 8 of these 22 (36.4%) patients with E2A-PBX1 fusion transcripts had K-ras mutations; 3 of the 22 (13.6%) patients had p53 mutations; only 1 of the 22 (4.5%) patient had EGFR mutation. K-ras and EGFR mutations in these patients were mutually exclusive to each other, same as previously reporeted [26]. Notably, 12 of the 22 (54.5%) patients had none of these three common mutations in lung cancer (Table 3 and Table 4). This subgroup of patients included all four non-smokers (patient # 4, 8, 13 and 22) and 8 stage I patients with AIS (Table 3). Among all thirteen NSCLC cell lines that we examined, only H1666 that was derived from a female patient with BAC (non-smoker) had no mutations in K-ras, p53 and EGFR (Note: the histology information of H1666 from ATCC still uses BAC). Taken together, our results suggest that the E2A-PBX1 fusion gene may be a genetic biomarker in NSCLC, especially in its subtype AIS.

**Table 3:** Mutational analysis of K-ras, p53 and EGFR in NSCLC patients with E2A-PBX1 fusion transcripts and NSCLC cell lines

**#**	**E2A-PBX1**	**k-Kras codon 12**	**p53**	**EGFR**	**Age**	**Gender**	**Race**	**Smoking status**	**Stage**	**Histology**
2	+				67	M	Middle Eastern	S	IIIB	Adc
12	+				72	F	Hispanic	S	IB	Adc
14	+				66	F	Caucasian	S	IIB	Adc
18	+	G12V			78	F	Caucasian	S	IB	Adc
20	+	G12C			63	F	Caucasian	S	IV	Adc
1	+				71	F	Caucasian	S	IA	AIS
3	+				41	M	Caucasian	S	IV	AIS
8	+				59	M	Caucasian	NS	IB	AIS
9	+				73	F	Caucasian	S	IIIB	AIS
11	+				84	M	Caucasian	S	IA	AIS
13	+				61	F	South Asian	NS	IB	AIS
21	+				82	F	Caucasian	S	IA	AIS
22	+				48	F	East Asian	NS	IB	AIS
16	+		G245S		63	F	Caucasian	S	IA	AIS
7	+		V272M	L858R	73	F	Caucasian	S	IIIB	AIS
6	+	G12C			68	M	Southeast Asian	S	IIIA	AIS
10	+	G12C			71	M	Hispanic	S	IA	AIS
17	+	G12C			67	F	Caucasian	S	IB	AIS
19	+	G12C			79	F	Caucasian	S	IIA	AIS
5	+	G12A			54	F	Caucasian	S	IIIB	AIS
15	+	G12V	exon 7(FS)		67	F	Caucasian	S	IV	AIS
4	+				39	M	Caucasian	NS	IB	LCC
23	+	ND	72	F	Caucasian	S	IA	AIS		
Cell line										
**A549**	+	G12S			58	M	Caucasian			Adc
**A427**	+	G12D			52	M	Caucasian			Adc
H441	-	G12V	R158L			M				Adc
H838	-				59	M	Caucasian	S	IIIB	Adc
H1975	-		R273H	L858R, T790M		F		NS		Adc
H1650	-			Exon 19 deletion	27	M	Caucasian	S	IIIB	Adc
H322	-		R248L		52	M	Caucasian			BAC
H358	-	G12C	Null			M	Caucasian			BAC
**H1666**	+				50	F	Caucasian	NS	III?	BAC
H2170	-		R158G			M		NS		Sqc
H1703	-		E285K		54	M	Caucasian	S	I	Sqc
H460	-	Q61H				M				LCC
H1299	-		Null		43	M	Caucasian			LCC

FS: frame shift; M: male; F: female; S: smoker; NS: non-smoker; Adc: adenocarcinoma; AIS: adenocarcinoma in situ; BAC: brochioloalveolar carcinoma (histology information from ATCC still uses BAC); LCC: large cell carcinoma; Sqc: squamous carcinoma; ND: not determined.

**Table 4:** Summary of mutational analysis in NSCLC patients with E2A-PBX1 fusion transcripts

		**Total (%)**	**K-P-E-**	**K + P-E-**	**K-P + E-**	**K + P + E-**	**K-P + E+**	**K-P-E+**	**K + P-E+**	**K + P + E+**
Total		**22 (100)**	12 (54.5)	7 (31.8)	1 (4.5)	1 (4.5)	1 (4.5)			
Gender	F	**15 (100)**	7 (46.7)	5 (33.3)	1 (6.7)	1 (6.7)	1 (6.7)			
	M	**7 (100)**	5 (71.4)	2 (28.6)						
Race	Caucasian	**16 (100)**	8 (50.0)	5 (31.3)	1 (6.3)	1 (6.3)	1 (6.3)			
	Asian	**3 (100)**	2 (66.7)	1 (33.3)						
	Middle eastern	**1 (100)**	1 (100)							
	Hispanic	**2 (100)**	1(50.0)	1 (50.0)						
Smoking status	NS	**4 (100)**	4 (100)							
	S	**18 (100)**	8 (44.4)	7 (38.9)	1 (5.6)	1 (5.6)	1 (5.6)			
Stage	I	**12 (100)**	8 (66.7)	3 (25.0)	1 (8.3)					
	II	**2 (100)**	1 (50.0)	1 (50.0)						
	III	**5 (100)**	2 (40.0)	2 (40.0)			1 (20.0)			
	IV	**3 (100)**	1 (33.3)	1 (33.3)		1 (33.3)				
Histology	AIS	**16 (100)**	8 (50.0)	5 (31.3)	1 (6.3)	1 (6.3)	1 (6.3)			
	Invasive Adc	**5 (100)**	3 (60.0)	2 (40.0)						
	LCC	**1 (100)**	1 (100)							

K: k-ras codon 12; P: p53 exons 4-8; E: EGFR exons 19-21.

## Discussion

Somatic genetic changes have been believed to play important roles in human tumorigenesis, but the cancer type in which somatic rearrangement occurs is limited to leukemias, lymphomas and soft tissue tumors [2]. Overexpression of Notch3 was found to be associated with chromosome 19 translocation in lung cancer [27]. EML4-ALK fusion gene [28] and ETS fusion genes [29,30] exist in NSCLC and prostate cancer, respectively. It is still unclear whether chromosome aberrations are important in the initiation of epithelial tumorigenesis.

AIS (formerly named BAC) is a subset of adenocarcinoma characterized by non-invasive growth along alveolar septae [19,25]. It is more prevalent in women, non-smokers, and Asians [25]. Despite the lack of stromal, vascular, or pleural invasion, AIS is malignant and surgical resection is currently the mainstay of curative treatment. We previously discussed about a multi-step model of lung cancer development, especially AIS as carcinoma in situ [31]. Genetic changes can sequentially accumulate and cause bronchioalveolar stem cells to transform, leading to development of invasive phenotype in human cancers. However, it is unclear what is the cause for transformation of atypical bronchioloalveolar cells into invasive adenocarcinoma or maintenance for the growth characterization in AIS. Several important players such as K-ras, p53, and survivin, etc. have been considered as tumor markers in AIS progression into invasive cancer [16-18,25], few are specific to AIS and “driver mutations” for AIS progression are still unidentified. Here in this study, we reported in NSCLC the expression of E2A-PBX1 fusion transcripts that have been well documented in leukemias [5-15]. This is the first report of detection of the E2A-PBX1 fusion transcripts in solid tumors. More interestingly, we observed that the E2A-PBX1 fusion transcripts were more frequently found in AIS than other subtypes of NSCLC, and the presence of E2A-PBX1 fusion transcripts were significantly associated with decreased overall survival in female and stage IA patients with AIS. These results suggest that the E2A-PBX1 fusion transcripts may play a critical role in AIS progression, especially for females and non-smokers. Supportive evidence also comes from our analysis of mutations in K-ras, p53 and EGFR that are common in NSCLC and considered as “driver mutations” [16-18]. Comparison of the mutational status of these genes in patients expressing the E2A-PBX1 fusion transcripts showed that approximately 55% patients examined in our study cohort were wild type in K-ras, p53 and EGFR. Majority of this subgroup were patients with AIS including all four non-smokers. Because E2A-PBX1 onco-protein has been proved to exhibit transformation potentials by transcribing target genes [5-15], we argue that E2A-PBX1 may serve as one “driver mutation” in AIS and play critical roles during initiation and progression of at least a subset of AIS. E2A-PBX1 may represent a new therapeutic target for NSCLC, especially AIS. Further investigation is needed to evaluate the function of E2A-PBX1 fusion protein, as well as its therapeutic and prognostic values and its correlation with treatment resistance in AIS.

In this study, we only examined in NSCLC specimens the conserved E2A-PBX1 fusion transcripts that are well documented in leukemias [5-15]. It is possible that other forms of E2A-PBX1 fusion transcripts also exist in NSCLC. TCGA (The Cancer Genome Atlas) data may be useful to analyze the frequency of E2A-PBX1 fusion transcriptions in NSCLC. Another limitation of this study is relatively small number of AIS specimens analyzed. Analysis of an independent large cohort of AIS is needed to validate our observation.

## Conclusions

Our data demonstrated the presence of E2A-PBX1 fusion transcripts caused by t(1;19)(q23;p13) in lung adenocarcinomas, especially AIS. It may be a common genetic change in AIS and a survival determinant for female AIS patients at early stage. These data may be of significant clinical importance, because finding reliable genetic biomarkers for early-stage lung adenocarcinomas including AIS is becoming increasingly apparent for early identification and management of this deadly disease.

## Consent

Written informed consent was obtained from the patient for publication of this report and any accompanying images.

## Competing interest

The authors declare that they have no competing interests.

## Authors’ contributions

MLM carried out the RNA extraction, primer design and PCR. TH carried out the DNA extraction and sequencing analysis. ZC and HL performed the statistical analysis. DJ participated in the design of the study. HMZ and BH conceived of the study, and participated in its design and coordination and helped to draft the manuscript. All authors read and approved the final manuscript.
